# Progress of research on PD-1/PD-L1 in leukemia

**DOI:** 10.3389/fimmu.2023.1265299

**Published:** 2023-09-26

**Authors:** Huizhen Cao, Tianyu Wu, Xue Zhou, Shuyang Xie, Hongfang Sun, Yunxiao Sun, Youjie Li

**Affiliations:** ^1^ Department of Pediatrics, Yantai Affiliated Hospital of Binzhou Medical University, Yantai, China; ^2^ Department of Gastrointestinal Surgery, Yantai Affiliated Hospital of Binzhou Medical University, Yantai, China; ^3^ Department of Biochemistry and Molecular Biology, Binzhou Medical University, Yantai, China

**Keywords:** leukemia, programmed cell death protein 1, programmed death-ligand 1, immunotherapy, PD-1/PD-L1 mAbs

## Abstract

Leukemia cells prevent immune system from clearing tumor cells by inducing the immunosuppression of the bone marrow (BM) microenvironment. In recent years, further understanding of the BM microenvironment and immune landscape of leukemia has resulted in the introduction of several immunotherapies, including checkpoint inhibitors, T-cell engager, antibody drug conjugates, and cellular therapies in clinical trials. Among them, the programmed cell death protein 1 (PD-1)/programmed death-ligand 1 (PD-L1) axis is a significant checkpoint for controlling immune responses, the PD-1 receptor on tumor-infiltrating T cells is bound by PD-L1 on leukemia cells. Consequently, the activation of tumor reactive T cells is inhibited and their apoptosis is promoted, preventing the rejection of the tumor by immune system and thus resulting in the occurrence of immune tolerance. The PD-1/PD-L1 axis serves as a significant mechanism by which tumor cells evade immune surveillance, and PD-1/PD-L1 checkpoint inhibitors have been approved for the treatment of lymphomas and varieties of solid tumors. However, the development of drugs targeting PD-1/PD-L1 in leukemia remains in the clinical-trial stage. In this review, we tally up the basic research and clinical trials on PD-1/PD-L1 inhibitors in leukemia, as well as discuss the relevant toxicity and impacts of PD-1/PD-L1 on other immunotherapies such as hematopoietic stem cell transplantation, bi-specific T-cell engager, chimeric antigen receptor T-cell immunotherapy.

## 1 Introduction

The current standard clinical treatment for leukemia, as a non-solid malignant tumor, mainly includes chemotherapy and hematopoietic stem cell transplantation (HSCT). However, the treatment process faces a series of problems such as chemotherapy insensitivity, chemoresistance, post-transplant relapse, and intolerance in elderly patients (1–4), thereby greatly limiting the progress of treatment for patients with leukemia. Therefore, developing effective methods with low adverse reactions is currently imperative to ameliorate the prognoses of leukemia patients. The immune milieu of bone marrow (BM) is dramatically altered in patients with leukemia, where tumor cells prevent themselves from being cleared by immune system by affecting suppressive immune responses (5–8). Moreover, tumor cells in the blood, BM, and lymphoid tissue are also more accessible to immune cells than solid tumors. Furthermore, the efficacy of allogeneic HSCT (allo-HSCT) demonstrates that leukemia is a typical immune-responsive tumor type (9). Thus, immunotherapy is an obvious choice for treating hematological malignant tumors. In hematologic tumors, currently used immunotherapies include allo-HSCT, bi-specific T-cell engager (BiTE), chimeric antigen receptor (CAR) T-cell immunotherapy (CAR-T), immune-checkpoint inhibitors (ICIs), and other monoclonal antibodies (mAbs) targeting tumor-cell surface antigens (10–13). In recent years, the role of immune escape in leukemia progression and development of immunotherapy have been elucidated, employing ICIs to block suppressor molecules on the surface of T cells, thereby reversing the “exhausted” state of T cells to an “activated” one to kill tumor cells, has proved to be a promising option.

Immune checkpoint (IC) is a signal regulating T-cell receptor (TCR) antigen recognition during immune response. Programmed cell death protein 1 (PD-1)/programmed death-ligand 1 (PD-L1) as an important IC modulating immune response. PD-1 (CD279), a type I transmembrane protein inhibitory checkpoint molecule is expressed on various immune cells, such as naive and activated B cells, effector T cells, regulatory T cells (Tregs), dendritic cells (DCs), activated monocytes, macrophages, natural killer (NK), and immature Langerhans cells (14). PD-1 receptors bind two ligands of the B7 family, PD-L1 and programmed death-ligand 2 (PD-L2). PD-L1 (CD274*)* is expressed on the surface of hematopoietic cells, such as DCs, macrophages, T cells, and B cells (15, 16). PD-L2 (PDCD1LG2) is expressed on monocytes, myeloid DCs, and activated CD4+ or CD8+ T-cell subsets (17). PD-L1 and PD-L2 differ in expression patterns but have the same effect, and binding of PD-1 to either ligand leads to T-cell dysfunction or exhaustion, resulting in diminished intensity of antigen-specific T-cell response in tumor tissues (18–21). In hematological malignant tumors, the expression rate of PD-L1 in malignant cells is 37%–58% (22). Leukemia cells highly express checkpoint-inhibitor receptors for sharing an immune-cell lineage (9, 23), making them potential targets for this therapy. This review centers around PD-1 signaling, summarizes its molecular functions in hematological malignant tumors and the achievements of ICIs in preclinical development and clinical settings.

## 2 Mechanisms involved in tumor immune escape through PD-1/PD-L1

**Figure 1:**
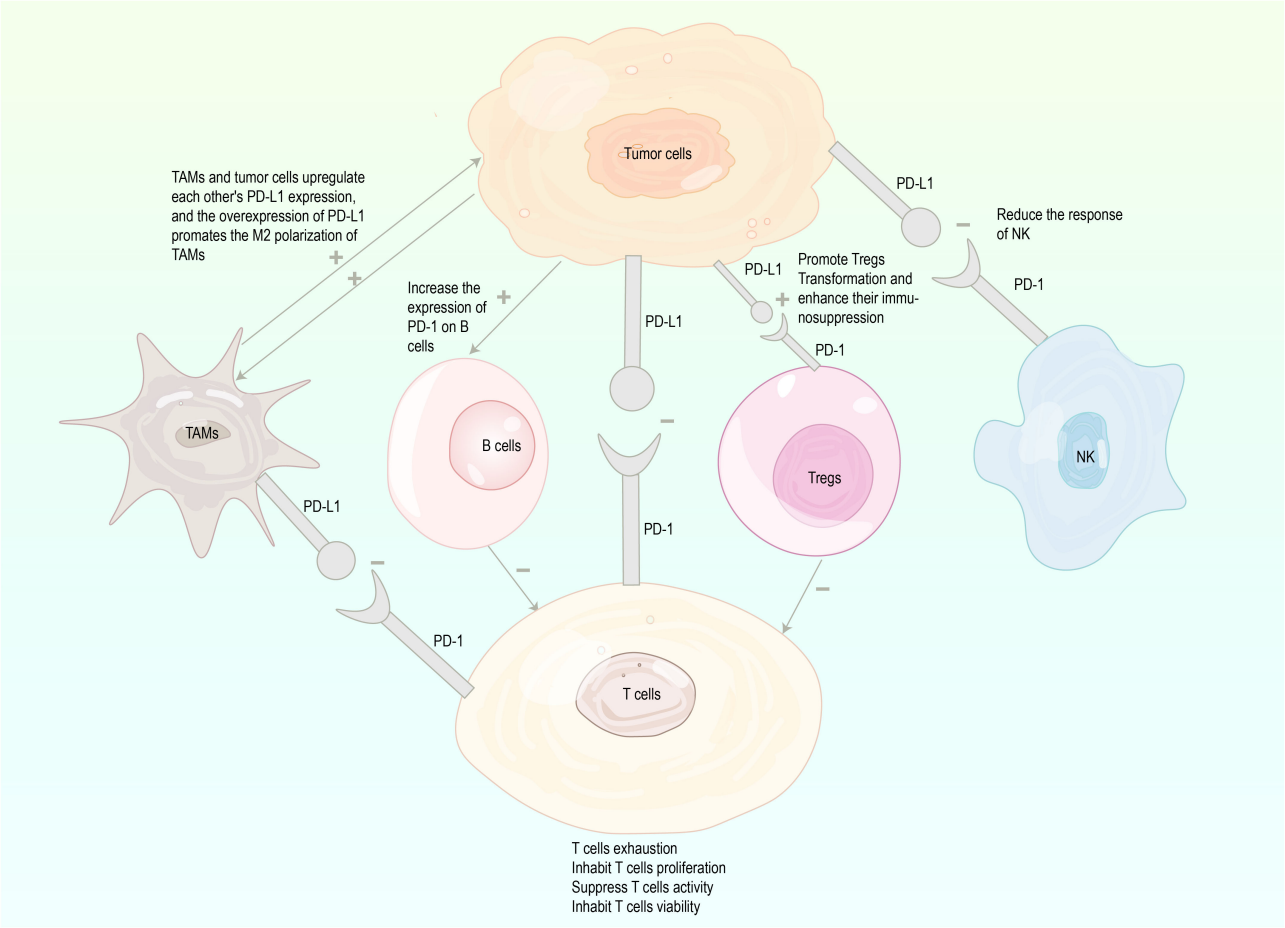
Mechanisms involved in tumor immune escape through the PD-1/PD-L1 (inhibition marked with -, enhancement marked with+), PD-1, programmed cell death protein 1; PD-L1, programmed death-ligand 1; TAMs, tumor associated macrophages; Tregs, regulatory T cells; NK, natural killer.

As a pair of co-stimulatory signals, PD-1 and PD-L1 jointly constitute PD-1/PD-L1 signaling pathway. Under physiological conditions, the binding of PD-L1 on cell surface to PD-1 on lymphocyte surface inhibits lymphocyte function and induces the apoptosis of activated lymphocytes. The activation of the PD-1/PD-L1 pathway reduces the damage of immunoreactions to surrounding tissues and prevents the progression of autoimmune diseases (24). However, the activation of this pathway causes the binding of PD-L1 expressed on tumor cells to PD-1 on tumor infiltrating lymphocytes, decreasing the immune effect of T cells in the local tumor microenvironment (TME), thereby mediating tumor immune escape and promoting cancer progression (25–27). Researches have shown that PD-L1 expression is upregulated in tumor cells, which activates PD-1/PD-L1 downstream pathways by specifically binding to PD-1 on the surface of cytotoxic T lymphocytes (CTLs) to deliver negative regulatory signals. In turn, it induces the exhaustion of activated T cells and the loss of immunoreactivity, leading to a diminished intensity of antigen-specific CTL responses in tumor tissues (18–21). Besides, Tregs as important suppressive immune cells in TME contribute to cancer initiation and progression. The PD-1/PD-L1 pathway promotes Tregs transformation and enhances their immunosuppressive capacity (28–30). In addition to T cells, other immune cells are implicated in the regulation of immune tolerance induced by the PD-1/PD-L1 pathway. Tumor-associated macrophages (TAMs) upregulate PD-L1 expression of tumor cells (31), whereas tumor cell-secreted versican and derived exosomes induce upregulation of PD-L1 expression in TAMs, which is associated with M2 polarization of TAMs. TAMs with high expression of PD-L1 more significantly inhibit effector T cells and promote tumor growth and metastasis (32–34). Tumor cells increase PD-1 expression on B cells (35, 36), and PD-1+ B cells significantly suppress the proliferation and reduce the viability of CD4+ and CD8+ T cells via the PD-1/PD-L1-dependent pathway (37). NK cells can obtain PD-1 from leukemia cells by endocytosis in tumor cells, and PD-L1 in tumor cells interacts with PD-1 of NK cells to reduce NK cell responses and produce more aggressive tumors (38–40) (**Figure 1**).

PD-1 signaling is a pivotal molecule mediating immune escape in TME. Blocking PD-1 signaling attenuates tumor cell suppression of immune cells and improves immune system recognition and cytotoxicity of tumor cells. The increasing understanding of immune function and immune escape mechanisms has led to exploitation of therapeutic mAbs targeting PD-1 signaling (25). Up to now, FDA has successively approved four mAbs (pembrolizumab, nivolumab, cemiplimab, and dostarlimba) targeting PD-1 and three mAbs (atezolizumab, avelumab, and durvalumab) targeting PD-L1 for the treatment of solid and hematological malignancies (15, 16, 41–46), as presented in **Table 1**.

**Table 1:** FDA approves mAbs for PD-1/PD-L1.

Drugs	Target	Timeline and cancer type
Pembrolizumab	PD-1	2014: Melanoma; 2015: NSCLC; 2016: HNSCC; 2017: Hodgkins Lymphoma, MSI-H or dMMR cancer, Gastric cancer, Bladder Cancer; 2018 Merkel cell carcinoma, Hepatocellular carcinoma, Cervical cancer, PMBCL; 2019: RCC, SCLC, Esophagus cancer; 2020: Colorectal cancer, Cutaneous squamous-cell carcinoma, TMB-high cancers; 2021: Breast cancer, Endometrial Carcinoma
Nivolumab	PD-1	2014: Melanoma; 2015: NSCLC, RCC; 2016: Hodgkins Lymphoma, HNSCC; 2017: Colorectal cancer; Hepatocellular carcinoma, Bladder Cancer; 2018: SCLC; 2020: Esophagus cancer, Malignant Pleural Mesothelioma; 2021: Gastric cancer
Cemiplimab	PD-1	2018: Cutaneous squamous-cell carcinoma;2021: NSCLC, Basal Cell Carcinoma
Dostarlimab	PD-1	2021: dMMR solid cancers, Endometrial Carcinoma
Atezolizumab	PD-L1	2016: NSCLC, Bladder Cancer; 2019: SCLC, Breast cancer; 2020: Melanoma, Hepatocellular carcinoma; 2022: ASPS
Durvalumab	PD-L1	2017: Bladder Cancer; 2018: NSCLC; 2020: SCLC; 2022: Hepatocellular carcinoma, Billiary track
Avelumab	PD-L1	2017: Merkel cell carcinoma, Bladder Cancer; 2019 RCC

FDA, Food and Drug Administration; mAbs, monoclonal antibodies; PD-1, programmed cell death protein 1; PD-L1, programmed cell death-ligand 1; NSCLC, non-small cell lung cancer; HNSCC, head and neck squamous cell carcinoma; MSI-H, high microsatellite instability; dMMR, deficient mismatch repair; PMBCL, Primary mediastinal large B-cell lymphoma; RCC, renal cell carcinoma; SCLC, small cell lung cancer; TMB, tumor mutational burden; ASPS, Alveolar soft part sarcoma.

## 3 Role of PD-1/PD-L1 in the development of leukemia

Leukemia can be divided four major clinical categories: acute myeloid leukemia (AML), chronic myeloid leukemia (CML), acute lymphoblastic leukemia (ALL), and chronic lymphocytic leukemia (CLL). This review provides a theoretical basis for drug discovery and clinical application of PD-1/PD-L1 pathway by summarizing and analyzing the role of PD-1 signaling in various types of leukemia.

### 3.1 AML

AML is a heterogeneous disease with various genetic and epigenetic alterations. Its pathogenesis is the accumulation and expansion of immature myeloid cells in the peripheral blood (PB) and BM, resulting in hematopoietic dysfunction. Historically, AML has been regarded as an immunoreactive malignancy and remains the most common indication to receive allo-HSCT (7). PD-1 expression is generally high on T cells in AML patients with *de novo* and relapsed/refractory (R/R) after chemotherapy, and partial recovery is achieved in patients with complete remission (47–49). Moreover, the level of PD-1 on NK cells and PD-L1 on regulatory B cells (Bregs) increases in AML patients (47, 48, 50, 51). High expression of PD-1 coincides with the T-cell exhaustion (52–54). The overexpression of PD-1 signaling is relevant to poor overall survival of AML patients (55). Above studies suggest PD-1 signaling may influence the development and poor prognosis of AML by increasing T-cell exhaustion. Contrary to this conclusion, Schnorfeil et al. (56) found the level of PD-1 expression on PB CD4+ and CD8+ T cells of AML patients at diagnosis was similar to that of healthy controls, but significantly increased in relapse after stem cell transplantation. T-cell function is not impaired during this process. They thought that this pattern is associated with a shift toward effector memory cells in patients with recurrent AML and T-cell exhaustion does not play a major role in AML. Besides, AML cells induce generation and expansion of Tregs by PD-1 signaling, and Tregs promote the proliferation of AML cells by secreting IL-10 and IL-35 (57, 58). In addition to regulating immune cells, PD-1/PD-L1 drives AML progression by regulating tumor-associated proteins, for example, the expression of PI3K and p-AKT decreases after PD-L1 knockdown, which induces G2/M cell cycle arrest and apoptosis, and the upregulation of PD-L1 increases the expression of PI3K/AKT and enhances the proliferation of tumor cells (59). PD-L1 is overexpressed in AML leukemia-initiating cells, where it increases cyclin D2 expression by enhancing JNK phosphorylation, ultimately promoting the entry of leukemia-initiating cells into cell cycle and proliferation (60). Epigenetic therapy (EGT), particularly with hypomethylating agents (HMAs) either alone or in combination, continues to be successfully used in treating elderly AML, although resistance is a frequent and ultimately near universal outcome (61). Liu et al. (62) found that EGT treatment induces the expression of PD-L1 mRNA and PD-L1 induces the occurrence of EGT resistance. To sum up, PD-1 signaling can promote AML progression by regulating immune cells, oncoproteins, and the occurrence of drug resistance, inhibition of PD-1 signaling can be a breakthrough for successful treatment of AML.

### 3.2 CML

CML is a myeloproliferative disorder characterized by BCR-ABL oncoprotein with high tyrosine kinase activity, which promotes the proliferation and inhibits the apoptosis of cancer cells (63). PD-1 signaling on specific T cells leads to T-cell exhaustion, and leukemia cells inhibit effector T-cell proliferation through PD-1/PD-L1 interactions, blocking PD-1 signaling contributes to improved CML control in pre-clinical mouse models by restoring the function of CML-specific CTLs (64). The quantity of bcr-abl fusion gene, as the initiation and core factor of CML pathogenesis, is positively correlated with the PD-1 expression level on CD8+ T cells. When CML is treated with tyrosine kinase inhibitors (TKIs), a target drug for bcr-abl, the PD-1 expression level of CD8+ T cells in the complete hematological response group is significantly lower than that in the control group, chronic phase, and blast phase (22). However, leukemia stem cells (LSCs) are resistant to specific TKIs and cause disease relapse after drug discontinuation in CML, besides, CTL transfer therapy leads to upregulation of PD-L1 on LSCs, which protects LSCs from CTL-mediated elimination. In contrast, PD-1 blockade during CTL transfer results in long-term survival of CML mice, suggesting that LSCs were either eliminated or effectively controlled by PD-1 blockade (65, 66). The Tregs are also increased in CML patients at diagnosis and in patients refractory to TKI treatment, and these Tregs have higher levels of PD-1 expression (67, 68). Which suggests PD-1-blocking antibodies given directly prior to and temporarily after TKI discontinuation may block the immune inhibitory effects of Tregs on CD4+/CD8+T-cells, blocking aberrant PD-1 signaling may result in greater success in TKI cessation studies.

### 3.3 ALL

ALL results from a clonal expansion of abnormal lymphoid progenitors of B cell (BCP-ALL) or T cell (T-ALL) origin that invades BM, PB, and extramedullary sites (69). Similar to other types of leukemia, PD-1 expression increases on T-cell subsets in B-ALL patients and is more prominent at relapse, PD-L1/L2 expression increases on LSCs (70). PD-1+ LSCs are used for T-ALL initiation and relapse, they can upregulate genes related to the MYC pathway, leukemic stemness, and early T-cell progenitor development, and downregulate genes related to apoptosis, cell cycle, and PI3K/AKT signal pathway (71). To determine whether PD-L1 expression on ALL cells inhibits T-cell responses, Blaeschke et al. (72) co-cultured second-generation anti-CD19 CAR-T cells with CD19+ and CD19+/PD-L1+ target cells. Result shows that CAR-T cells co-cultured with PD-L1+ target cells decrease the levels of Th1 cytokine secretion. Which indicates that PD-1 signaling mediates T-cell inhibition after/during T cells against BCP-ALL. In summary, above studies suggest that enhancing T-cell response by inhibiting PD-L1/L2 is a promising therapeutic option.

### 3.4 CLL

CLL is characterized by the accumulation and clonal proliferation of mature and typically CD5+CD23+ B-cells within PB, BM, lymph nodes, and spleen (73). Several studies have shown that PD-1 signaling is significantly upregulated in CLL patients, and the high level of PD-1/PD-L1 is closely related to disease grade and poor prognosis (74–78). Epstein–Barr virus (EBV) is one of the human tumor viruses, it can transform B-cells into tumor cells. In CLL patients, EBV load is positively correlated with the expression of PD-1 signaling on CD4+ and CD8+ T cells. In EBV (+) patients, the higher the level of PD-1 signaling on T cells, the higher the risk of lymphocyte doubling and treatment initiation (79). Gassner et al. (80) found that inhibiting the interaction of PD-1/PD-L1 can reactivate the cytotoxic effect of exhausted T cells in CLL mouse model. To study the mechanism of PD-1 signaling in CLL, Qorraj et al. (81) collected PB mononuclear cells from CLL patients. They found that triggering PD-1 on monocytes hampers phagocytosis, glycolysis, and Bruton’s tyrosine kinase (BTK)-signaling. Conversely, the immune metabolic dysfunctions and antitumor activity of monocytes can be reversed by disrupting PD-1 signaling. In conclusion, PD-1 signaling inhibits immune cell activity and interferes with immune metabolic processes. The blockade of PD-1 signaling may improve the prognosis of CLL.

## 4 Regulation of the PD-1/PD-L1 pathway in leukemia

**Figure 2:**
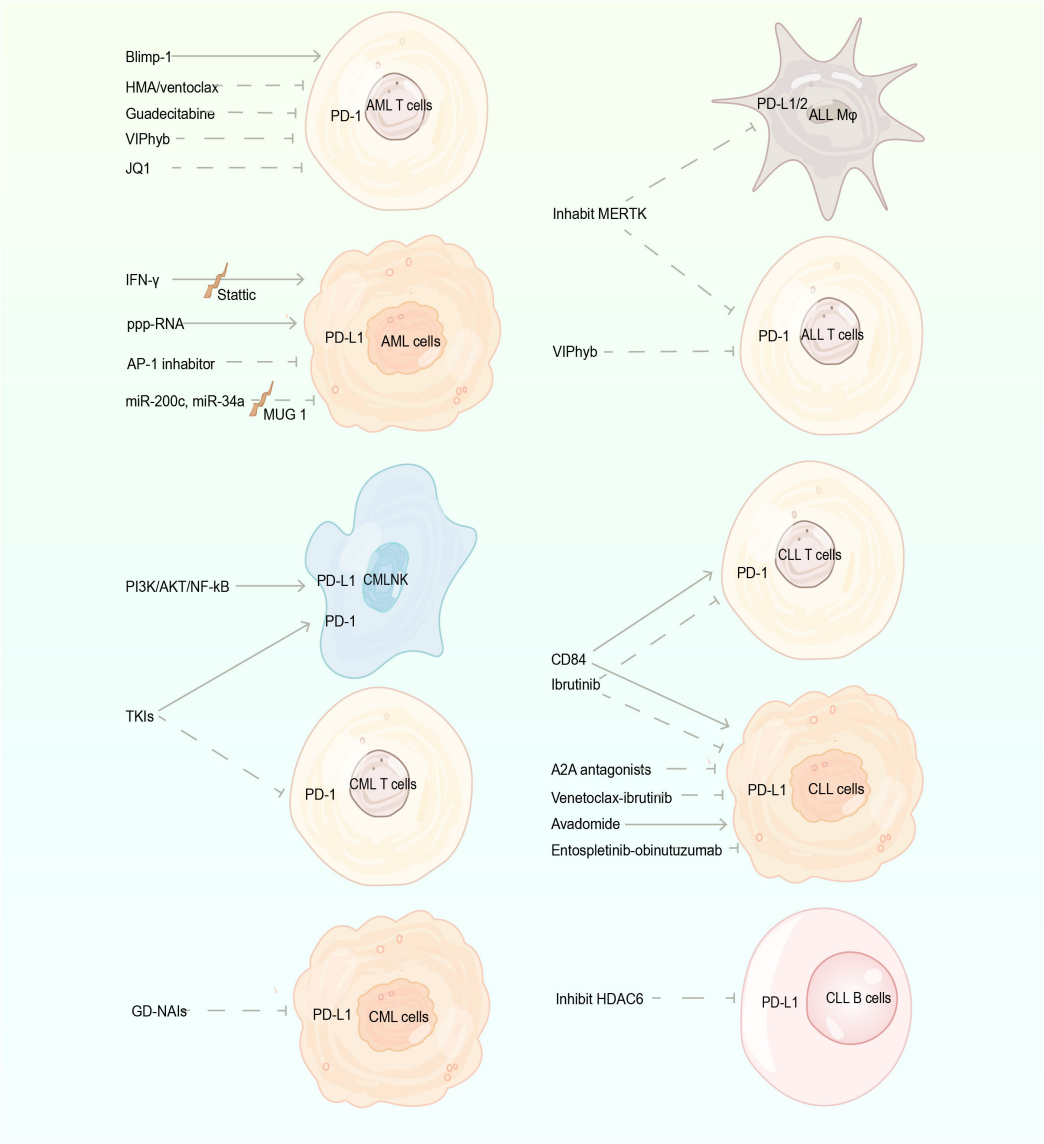
The regulation of the PD-1/PD-L1 pathway. PD-1, programmed cell death protein 1; PD-L1, programmed death-ligand 1; AML, acute myeloid leukemia; ALL, acute lymphoblastic leukemia; CML, chronic myeloid leukemia; CLL, chronic lymphocytic leukemia; Blimp-1, B lymphocyte-induced maturation protein 1; HMA, hypomethylating agents; TKIs, tyrosine kinase inhibitors; GD-NAIs, *Gynura divaricate*-non-alkaline ingredients; Mφ, monocytes/macrophages; A2A, Adenosine A2A receptor.

In addition to PD-1 and PD-L1 antibodies directly acting on PD-1 signaling, other proteins, genes, and drugs affect the level of PD-1/PD-L1. When mAbs are insensitive or patients are intolerant to adverse reactions, we may consider indirectly inhibiting immune escape of tumor cells by regulating related proteins and genes or applying relevant drugs (**Figure 2**).

### 4.1 AML

In AML patients, B lymphocyte-induced maturation protein 1 (Blimp-1) directly binds to the promoter of PD-1 and impairs T-cell activity by upregulating PD-1. The knockdown of Blimp-1 can reverse the T-cell functional defect (82). IFN-γ induces PD-L1 expression in myeloid precursor cells and primary cells (57, 83). Stattic, a small molecule inhibitor of STAT3, interferes with IFN-γ-induced PD-L1 expression in AML (84). PD-1 level decreases after the initial HMA/ventoclax (Bcl-2 inhibitor) treatment on all CD4+ T-cell subpopulations except naïve in AML patients (48). In an immunocompetent murine leukemia model, guadecitabine (a second-generation HMA) negatively regulates inhibitory accessory cells in TME by reducing PD-1+ T cells and the AML-mediated expansion of myeloid-derived suppressor cells. Consequently, functionally active leukemia specific T cells increase (85). NA-AML (Nras^G12D/-^; Asxl^-/–^AML) cells overexpress PD-L1/PD-L2, and the level of PD-L1 is associated with the upregulation of AP-1 transcription factor (TF). AP-1 inhibitor or short-hairpin RNAs against AP-1 TF Jun decreases PD-L1 expression (86). The overexpression of miR-200c and miR-34a causes the significant downregulation of PD-L1 level. MUC1 attenuates the interference of miR-34a and miR-200c on PD-L1 translation by negatively regulating the expression of miR-34a and miR-200c, and silencing of MUC1 leads to increased miR-34a and miR-200c. In turn, PD-L1 expression is reduced (87).

### 4.2 CML

Myeloid leukemia cells induce PD-L1 expression on NK cells via PI3K/AKT/NF-kB pathway (88), thus, inhibiting this pathway may block PD-L1 expression. The level of PD-1 on CD8+ T cells is reduced in CML patients treated with TKIs dasatinib and imatinib (22). *Gynura divaricata* (L.) DC. is a widely used herbal medicine, whose non-alkaline ingredients regulate PD-1 signaling, significantly inducing apoptosis and inhibiting proliferation of CML cells (89).

### 4.3 ALL

A leukemic microenvironment supports the survival of ALL cells and their immune evasion through multiple interactions (69). In an ALL mouse model, inhibition of MERTK significantly decreases the expression PD-L1/L2 on CD11b+ monocytes/macrophages and PD-1 on CD4+ and CD8+ T cells in the leukemic microenvironment, reducing the incidence of splenic FOXP3+ Tregs at sites of leukemic infiltration. Consequently, T-cell activation increases, and immune-mediated ALL clearance is promoted (90). Murine models of AML and T-ALL reveal that VIPhyb, a peptide antagonist of VIP signaling, enhances IFN-γ secretion and suppresses PD-1 expression in CD4+ and CD8+ T cells (91).

### 4.4 CLL

CD84-mediated intercellular interactions upregulate the level of PD-1 on T cells and PD-L1 on CLL-cells via the Akt-mTOR pathway, resulting in T-cell exhaustion. Conversely, the downregulation of CD84 expression reverses these phenomena and reduces the expression level of other exhaustion markers (92). The activation of Adenosine A2A receptor (A2A) induces immune tolerance and is closely associated with immune escape of tumor cells (93). In CLL cells, hypoxia causes the emergence of a population of PD-1+ and IL-10–secreting T cells, and adding A2A antagonists attenuates Tregs generation, TGF-β induction, PD-1 expression, and IL-10 synthesis and secretion. Thus, leukemia cells become more susceptible to pharmacological agents while restoring immune competence and T-cell proliferation (94). Ibrutinib, a covalent inhibitor of BTK, is approved for treatment of patients with R/R or treatment-naive CLL (95). Cubillos et al. (96) found that ibrutinib can decrease PD-1 and PD-L1 expression by driving Th1-selective pressure in T cells. Kondo et al. (95) suggested that ibrutinib enhances antitumor immune responses by inhibiting STAT3-induced selective and persistent downregulation of PD-L1 on CLL cells and PD-1 in CD4+ and CD8+ T cells. In a venetoclax (VEN)–ibrutinib combination treatment, the number of PD-1+CD8+ T cells, Tregs, and follicular helper T cells decreases more than fivefold, thereby reducing the immunosuppressive characteristics of CLL (97). The SYK inhibitor entospletinib in combination with obinutuzumab downregulates the expression of PD-1 in CD4+ and CD8+ T-cell subsets of CLL patients, partially reversing the T-cell exhausted phenotype (98).

## 5 PD-1/PD-L1 and allo-HSCT

Allo-HSCT is a potentially curative therapy for various hematologic malignancies. It relies on the graft-versus-leukemia (GVL) effect mediated by donor-derived alloreactive T cells. However, graft-versus-host disease (GVHD) is also mediated by the same T cells and remains a major clinical problem related to considerable morbidity and mortality (99, 100). The occurrence of GVHD and T-cell suppression is positively correlated with the expression level of PD-L1 (101). The loss of GVL effect is relevant to PD-1 overexpression in allograft recipients, and blocking PD-L1 largely restores GVL efficacy without triggering GVHD (102). Besides, HSCT leads to differential upregulation of PD-1 ligands in tissues, which compartmentalizes CTL activity and thus creates niches for tumor escape. PD-1 blockage can restore CTL sensitivity to antigens and homogenize the effect of graft against tumor (103). These suggest that improving GVL and reducing GVHD by blocking PD-1 signaling can yield considerable results. Ni et al. (100) found that the exhaustion of CD4+ T cells leads to PD-L1 upregulation in donor CD8+ T cells and recipient tissues. which increased PD-L1/PD-1 interplay between donor CD8+ T cells and recipient tissues contributes to preventing GVHD by promoting the apoptosis and exhaustion of T-cell in GVHD target tissues, and enhanced PD-L1/CD80 interplay between CD8+ T cells contributes to retaining GVL responses by improving T-cell expansion and survival. Accordingly, the influence of the PD-L1-mediated effect on HSCT depends on the tissue microenvironment, the existence of CD4+ T cells, and the natural interacting partner expressed by CD8+ T cells. This suggests that we can enhance the PD-1 signaling-mediated GVL effect and reduce the PD-1 signaling-mediated GVHD by changing the above conditions. Besides, VIPhyb also increases the anti-leukemic effect after allogeneic BM transplantation by downregulating PD-1 and PD-L1 expression on donor immune cells (104). In clinical trial, Tschernia et al. (105) found that the use of pembrolizumab before allo-SCT reduced 100-day mortality in AML patients (17% vs 0%) and did not increase grade III-IV acute GVHD. The chronic GVHD is not found in patients who have received pembrolizumab before allo-SCT and cyclophosphamide after transplantation. This suggests that ICI treatment prior to allo-SCT is effective and safe, and post-transplant cyclophosphamide can eliminate the GVHD risk and severity. The above studies provide an empirical and theoretical basis for ICIs combined with HSCT in the treatment of leukemia.

In addition to utilizing the GVL effect of hematopoietic stem cells (HSCs), Hu et al. (106) enhanced the delivery of checkpoint inhibitors by using the *in situ* activation of platelets and the homing ability of HSCs. They constructed HSC-platelet-aPD-1 conjugates and then injected them into mice bearing AML cells, the therapeutic effect of checkpoint blocking is significantly enhanced. With regard to the drug-delivery mode of PD-1/PD-L1, Chen et al. (107) introduced a transdermal cold atmospheric plasma (CAP)-mediated IC blockade (ICB) therapy. The ICB delivered via microneedles enhanced the immune response mediated by T cells. Han et al. (108) used HEK293T-derived vesicles with PD-1 receptors on their surface to destroy PD-1 signaling, while the internal space of the vesicle allows for the packaging of an indoleamine 2,3-dioxygenase inhibitor, which further enhanced the antitumor effect. This suggests that in addition to drug development for ICIs, new technologies for applying ICIs are also of interest worthy of attention.

## 6 Efficacy of PD-1/PD-L1 mAbs treating leukemia alone or in combination

Studies on leukemia treatment with PD-1/PD-L1 mAbs are rapidly increasing in number. They are primarily divided into basic research and clinical stages. Herein, we provide guidance and rationale for subsequent clinical applications by analyzing their pooled data.

### 6.1 PD-1/PD-L1 mAbs for AML

Current clinical treatments for AML are primarily chemotherapy and allo-HSCT. However, due to the emergence of resistance to chemotherapy and GVHD, more effective and safer drugs to treat AML need to be developed (109, 110). Nivolumab, a PD-1 mAbs, is applied in an index case of recurrent myeloproliferative neoplasms after HSCT. Before infusion of nivolumab, AML blasts show high expression of chemokines, whereas T cells are characterized by the expression of interferon-responsive genes. This baseline inflammatory signature disappears after infusion of nivolumab, and the clinical responses are characterized by the temporary expansion of polyclonal CD4+ T-cell populations, the contraction of AML subsets exhibiting megakaryocytic characteristics, and elevated PD-L1 expression (111). Several studies show that the combination of PD-1/PD-L1 mAbs is promising research, for instance, the combined blockade of PD-1 signaling and Tim-3 have an additive effect on inhibiting tumor growth in advanced AML mouse models (112). The combination of IL-15 and PD-1 blockers activates AML-NK cells and enhances the killing ability of NK by increasing the release of perforin, granzyme, and IFN-γ (113). In addition, inhibiting the effects of other therapies on PD-1 expression can also yield considerable results, for instance, exogenous short 5′-triphosphate-modified RNA (ppp-RNA) can direct the immune response toward tumor cells. However, ppp-RNA treatment induces PD-L1 expression on AML cells and establishes therapeutic sensitivity to anti-PD-1 *in vivo*, the combination of anti-PD-1 and ppp-RNA is superior to either regimen alone in the survival rate of a mouse model (114). The DAC/VEN therapy (HMA decitabine combined with BCL‐2 inhibitor venetoclax) effectively targets leukemia cells while upregulating PD-1 expression in AML patients. Nivolumab combined with DAC/VEN can enhance antitumor effect and eliminate circulating blasts and LSCs/progenitor cells (115). The above studies indicate that considering PD-1/PD-L1 antibodies as an adjuvant treatment scheme for AML can effectively enhance the sensitivity of cell therapy and chemotherapeutic agents, which is a promising combination-chemotherapy option. A number of clinical studies have been conducted on PD-1 mAbs combined with other chemotherapeutic drugs in the treatment of AML, such as cytarabine (116), azacytidine (117), decitabine (118), and these treatment regimens are clinically feasible and have shown encouraging results.

Tumor progression leads to increased Tregs and elevated PD-1 expression on CD8+ CTLs in AML mouse model, which reduces the recognition and activation of tumor-specific CTLs (58). PD-L1 siRNA-mediated silencing augments the expression of T cell activation markers (CD69 and CD137) and improves CTL degranulation (CD107a) (119). CTL infusion combined with PD-1 blockade suppresses Tregs (120). PD-1 blockade in combination with Tregs exhaustion or CTL infusion induces significantly more AML tumor reduction than either treatment alone (58, 120). Additionally, combining DC-based immunotherapy with PD-1 blockade might be a promising approach to eliminating LSCs (121). In sum, PD-1/PD-L1 blockade combined with cell therapy represents a significant new approach that can be easily translated into clinical applications to enhance T cell-mediated cytotoxic responses.

### 6.2 PD-1/PD-L1 mAbs for CML

TKIs and HSCT are the mainstay of treatment for CML (122, 123), and immune mechanisms may help maintain treatment-free remission. The direct interplay between NK cells and K562 myeloid leukemia cells induces the PD-L1 expression of NK cells. Compared with PD-L1-NK cells, PD-L1+ NK cells are activated effector cells with strong killing activity against tumor cells *in vitro*. The binding of the PD-L1 mAbs atezolizumab to PD-L1 upregulates PD-L1 expression on the surface of NK cells and provides more binding sites for PD-L1 mAbs, resulting in continuous activation of p38. This phenomenon further propagates strong activation signals toward NK cells to maintain their cytotoxic and cytokine-secretion features. *In vivo*, the combination of PD-L1 mAbs and NK cell-activating cytokines significantly enhances the antitumor activity of NK cells against myeloid leukemia lacking PD-L1 expression (88). This finding suggests that PD-L1 mAbs have a unique therapeutic effect on PD-L1- tumors, this is independent of PD-1. Dasatinib, a second-generation TKI, upregulates PD-1 expression on CD56^dim^NK cells and increases dysfunctional CD56^neg^NK cells that highly express PD-1. Nivolumab enhances the cytotoxic activity of both subsets but more efficiently in the CD56^dim^ subset compared with the CD56^neg^ subset (39). Which suggests the combination of TKIs and PD-1/PD-L1 mAbs may be an approach for the successful treatment of CML patients. Recent evidence shows PD-1 expression on CD4+ and CD8+ T-cells, including on CML-reactive PR1-CTL in TKI-naive but also TKI-treated remission CML patients (124–126), which suggests T-cell exhaustion also in deep molecular remission, this provides a rationale for the treatment with checkpoint blocking antibodies to PD-1/PD-L1. However, a clinical trial of the combination of dasatinib and nivolumab for the treatment of CML showed that this approach did not show meaningful clinical activity in patients with CML in chronic phase or accelerated phase who received ≥2 prior TKIs with progression, resistance, or suboptimal response to most recent therapy (127). A phase II trial of the effectiveness of pembrolizumab and dasatinib, imatinib mesylate, or nilotinib in treating patients with CML and consistently detecting minimal residual disease (defined as the level of a gene product called bcr-abl in the blood) is currently underway (www.clinicaltrials.gov as # NCT03516279).

### 6.3 PD-1/PD-L1 mAbs for ALL

ALL has genetic heterogeneity, and the incidence is much higher in children. The current therapies for ALL are primarily multidrug chemotherapy, which has a high response rate but also has a high recurrence rate, leaving much room for improvement (128, 129). The phenotypic exhaustion of CD4+ T-cells predicts recurrence and poor overall survival in B-ALL. In a Ph+ B-ALL mouse model, the application of PD-L1 antibody clonally expands leukemia-specific CD4+ T-cells with helper/cytotoxic phenotype and reduces the expression of exhaustion markers. The combination of PD-L1 mAbs and TKI nilotinib also significantly improves the efficacy of nilotinib against BCR-ABL+ B-ALL (130). Axl ablation in macrophages can elicit the susceptibility of PD-1 refractory treatment naive B-ALL to PD-1 checkpoint blockade and promote antileukemia immunity (131). A new peptide, nABPD1, is designed to specifically bind PD-1. It enhances cytokine-induced killer (ICIK) cell-mediated antitumor activity by protecting ICIK cells through blockade of PD-1 signaling (44). There is a lack of clinical studies on the use of PD-1/PD-L1 mAbs in ALL, especially in young people, and as far as the current studies are concerned, they show unsatisfactory results for MRD in adults(median age is 52.5y) with ALL (132). Two studies of PD-1 mAbs for the treatment of ALL in children, adolescents, and young adults are currently underway (www.clinicaltrials.gov as # NCT05310591, NCT04546399).

### 6.4 PD-1/PD-L1 mAbs for CLL

Chemotherapy and anti-CD20 mAbs therapy are the standard of care for patients with CLL (133–135). Currently, it is prominent to improve the complete remission rate and reduce chemotherapy-induced immunosuppression. Studies suggest that early blockade of PD-L1 effectively prevents the immune dysfunction induced by tumor cells and thus avoids CLL development in mice. This includes the prevention of exhaustion-like and aberrant T-cell phenotypes, and the restoration of MHC class II-expressing dendritic cells and mature macrophages (108, 136). Ioannou et al. (137) concluded that although PD-L1 mAbs are superior to PD-1 mAbs in inducing anti-CLL T-cell activity, PD-1 mAbs and PD-L1 mAbs monotherapies are largely ineffective in overcoming T-cell tolerance in CLL. Avadomide is a cereblon E3 ligase modulator drug that stimulates T-cell immune synapse while increasing PD-L1 expression, it triggers IFN-driven T-cell responses and converts noninflamed CLL tumors into CD8+ T cell-inflamed ones, making CLL sensitive to PD-1/PD-L1 immunotherapy. The combination of avadomide and PD-1/PD-L1 blockade effectively reinvigorates previously exhausted patient T cells and contributes to more T cell killing in CLL. HDAC6 gene silencing or inhibition decreases PD-L1 expression on B cells of Eµ-TCL1 mice model, and the combination of HDAC6 inhibitor ACY738 and anti-PD-1/anti-PD-L1 further enhances the cytotoxicity of T cells (138). Rivas et al. (139) found that the treatment of CLL with anti-PD-L1 in combination with IL-10 produces more IFN-γ+, memory CD8+ T-cells, and cytotoxic effector KLRG1+, and fewer exhausted T-cells than anti-PD-L1 alone. CLL animal experiments show that PD-1/PD-L1 antibody as a combination chemotherapy regimen definitely affects tumor inhibition. However, according to the results of the current clinical study, PD-1 mAbs have limited efficacy in CLL patients, but reassuringly they show a promising therapeutic option in patients with Richter’s transformation (140–142).

### 6.5 PD-1/PD-L1 mAbs and CAR-T

CAR-T cell therapy has contributed to a revolution in the therapy of patients with hematological malignancies (143). However, the activation of CAR-T cells can lead to persistently high levels of PD−1 and eventually cause the exhaustion of T cells (144). Several studies have shown that the integration of PD-1-mediated inhibitory signaling into CAR-T significantly improves the function of conventional CAR-T, and it even may have an almost equivalent or better anti-tumor effect and a lower side effect compared with the CAR-T plus PD-1 antibody (72, 145, 146). More studies on PD-1 signaling with CAR-T are shown in **Table 2**. These studies suggest that PD-1 signaling blockade combined with CAR-T can enhance the efficacy of CAR-T. To date, a variety of CAR-T with PD-1 inhibition have been designed, and they have achieved gratifying results in preclinical studies. PD-1 signal blocking combined with CAR-T may produce greater benefits compared with chemotherapeutic drugs. However, there is a lack of clinical studies in this area, and the clinical effects and adverse effects are unclear.

**Table 2:** CAR-T combined with PD-1/PD-L1 for leukemia treatment.

Condition	CAR-T product	Design	Phase	Outcome
(72) ALL	CD19 CAR-T, CD22 CAR-T	Anti-CD19 and anti-CD22 CAR T cells combined with PD-1-CD28 fusion protein	Preclinical trial	Increase function of CAR-T cells against leukemia and protect CAR-T cells from leukemia-induced suppression
(147) CLL	CD19 CAR-T	-	Clinical trial	The percentage of CAR-T cells with CD8+PD-1+ phenotype is significantly lower in complete-remission patients compared with partially responding and nonresponding patients.
(146) CML	CD19/△PD-1 CAR-T	Integrate PD-1 shRNA into a third-generation CAR plasmid	Preclinical trial	Suppress the immunosuppression of TME and prolong the activation time of CAR-T cells
(145) CML	aPDL1-CART	Integrate a PD-L1-targeted scFv fusion protein into a CAR	Preclinical trial	Successfully prevent the development of PD-L1-expressing leukemia xenografts in immunocompromised mice
(144) AML	CLL-1 CAR-T	Silence the expression of PD-1 in CLL-1 CAR-T	Preclinical trial	The killing ability of CLL-1 CAR-T is further enhanced
(148) AML	CD19-CAR-T, CD123-CAR-T	CAR-T treated with JQ1 [JQ1(BET inhibitors) can suppress PD-1 expression in T cell]	Preclinical trial	The antileukemia potency and anti-exhaustion ability of CAR-T cells are enhanced
(149) R/R AML	CLL-1 CAR-T	CLL-1 CAR-T cells with PD-1 knockdown in 2 patients	Clinical trial	Both patients achieved molecular complete remission with incomplete hematologic recovery at 28 days
(150) ALL	PD-1-CD28 IFP CAR-T	Fuse different variants of the extracellular domain of PD-1 to the intracellular domain of CD28 to create multiple variants in the protein length of the PD-1-CD28 IFP	Preclinical trial	IFP variants with physiological PD-1 length ameliorate CAR T cell effector function and proliferation in response to PD-L1+ tumor cells *in vitro* and prolonged survival *in vivo*

CAR-T, chimeric antigen receptor T-cell immunotherapy; ALL, acute lymphoblastic leukemia; PD-1, programmed cell death protein 1; CLL, chronic lymphocytic leukemia; CML, chronic myeloid leukemia; shRNA, short hair RNA; TME, tumor microenvironment; PD-L1, programmed death-ligand 1; CLL-1, C-type lectin-like molecule-1; BET, bromodomain and extra terminal domain BET; IFP, immunostimulatory fusion protein.

### 6.6 PD-1/PD-L1 mAbs and BiTE

Blinatumomab (BiTE antibody) is a novel immunotherapy that recruits the forces of T cells and guides them against lymphoblastic cells by binding CD3 expressed on the surface of T cells and CD19 expressed on the surface of B cell lines (151, 152). It was approved by FDA in 2014 for the treatment of Ph-negative R/R precursor B-ALL. However, approximately 50% of R/R B-ALL patients do not respond to blinatumomab. Non-responders consistently express higher levels of PD-1 during blinatumomab treatment, and the levels of PD-L1 and PD-L2 increase on residual tumor cells in BM after treatment. The T-cell responses of blinatumomab against leukemia are potentiated by blocking CTLA-4 and PD-L1 signaling pathways (153, 154). This finding illustrates that the response of blinatumomab is correlated with the molecular level of IC. Wunderlich et al. (155) reported that pembrolizumab combined with blinatumomab increases the clearance of B-ALL in mice and reverses T-cell lymphopenia induced by blinatumomab. PD-1 inhibition also enhances the efficacy of blinatumomab in a UCB/PDX model of recurrent pediatric B-ALL. Krupka et al. (156) constructed the CD33/CD3 BiTE antibody AMG 330. They found that PD-L1 on primary AML cells is strongly upregulated after adding AMG 330 in the ex vivo culture, and blocking PD-1/PD-L1 axis enhances the AMG 330-induced lysis of AML cells by reversing T-cell-induced immune escape. Herrmann et al. (157) fused the extracellular domain of PD-1 (PD-1ex), which naturally holds a low affinity to PD-L1, with an αCD3.αCD33 BiTE^®^-like scaffold to form a bifunctional checkpoint inhibitory T-cell-binding (CiTE) antibody. The CiTE antibody is more potent in binding to AML cells and T cells, thereby increasing the function of T-cell effectors, and minimizing iRAEs associated with the systemic application of ICB. From the above several ex vivo studies and animal experiments, we can conclude that BiTE and PD-1 signaling blockade have good synergy in leukemia treatment. Nevertheless, there are few completed clinical studies on the combination therapy of blinatumomab and PD-1/PD-L1 mAbs for leukemia. A large sample phase II trial comparing blinatumomab alone to blinatumomab with nivolumab in patients with relapsed B-ALL is currently underway (www.clinicaltrials. as # gov NCT04546399).

### 6.7 Clinical trials of PD-1/PD-L1 mAbs in leukemia

A number of clinical trials on leukemia treatment with PD-1/PD-L1 mAbs have been performed nowadays. The overall response rate (ORR) of pembrolizumab alone is 0% in eight patients with AML (158). When combined with cytarabine, the ORR is 46% (116); the former grade 3–4 iRAEs are 25% (158), and the latter grade ≥3 iRAEs are 14% and self-limiting (116). The median recurrence-free survival (RFS) of AML patients treated with nivolumab alone is 8.48 months (159). When combined with cytarabine–idarubicin, the RFS is 18.54 months (160); the former grade 3–4 iRAEs are 27% (159), and the latter are 13.6% (160). From the above data, we assume that the efficacy of pembrolizumab and nivolumab alone is significantly lower than that of the combination, and the incidence of iRAEs is also higher with the single agent than with the combination. The median overall survival (mOS) is 11.1 months for AML with 200 mg of pembrolizumab in combination with 1.5–2 g/m^2^ cytarabine (116), 21 months when combined with 1.5–2 mg/m^2^ cytarabine (105), and 10.8 months when combined with decitabine (118). Regarding the data from the current sample, the efficacy of pembrolizumab combined with low-dose cytarabine is superior to that of the high-dose one, and the efficacy of pembrolizumab combined with decitabine or high-dose cytarabine is similar. The iRAEs are 42% in AML patients treated with pembrolizumab alone, the grade 3–4 iRAEs are 25% (158), the iRAEs are 40% when treated with nivolumab alone, the grade 3–4 iRAEs are 27% (159), and the incidence of adverse events is similar for both drugs. The mOS for AML treated with avelumab–azacitidine combination is 4.8 months (161), and that with durvalumab–azacitidine combination is 13.0 months (162). The ORR for AML treated with avelumab–azacitidine combination is 10.5% (161), that with nivolumab–azacitidine combination is 33% (117), and that with durvalumab–azacitidine combination is 31.3% (162). However, the grade 3–4 iRAEs in the avelumab combination are less than 7.7% (163). Based on the current sample alone, avelumab is less effective than pembrolizumab, nivolumab, and durvalumab, but its incidence of iRAEs is much lower. Due to differences in sample size and patient disease status among studies, comparisons of efficacy and adverse effect assessments of PD-1/PD-L1 mAbs among studies are subject to large errors. Regarding the current sample, PD-1/PD-L1 mAbs are effective in the treatment of leukemia, but the effect of single drug therapy is weak, and the effect of combination is more considerable. The occurrence of iRAEs is also not negligible, and large sample data are required to clarify the curative effects and adverse effects of PD-1/PD-L1 mAbs. Studies on PD-1/PD-L1 mAbs in CML, ALL, and CLL are few, and more details about clinical trials on PD-1/PD-L1 mAbs in the treatment of leukemia are shown in **Table 3**.

**Table 3:** Clinical trials of PD-1/PD-L1 mAbs in leukemia.

Study population	Number (n)	ICIs	Target	Study design	Therapy regimen	Clinical benefits	iRAE
(116)R/R AML	37	Pembrolizumab	PD-1	Phase II, open-label, single-arm,	Pembrolizumab 200 mg after 1.5–2 g/m^2^ cytarabine	ORR 46% CRc rate 38% mOS 11.1 months	Grade ≥3 iRAEs are 14% and self-limiting
(118)R/R AML	10	Pembrolizumab	PD-1	Open-label, single-arm, single-institution	Pembrolizumab 200 mg on day 1 of every 3-week cycle, with decitabine 20 mg/m^2^ on days 8–12 and 15–19 of alternative cycles starting with cycle 1.	mOS 10.8 months	iRAEs are 30%
(105)R/R AML	9	Pembrolizumab	PD-1	Phase II, retrospective matched cohort	Cytarabine 1.5–2 mg/m^2^ every 12 hours days 1–5 followed by pembrolizumab 200 mg on day 14, every 3 weeks for up to 2 years	mOS 21 months 1-year RFS 44% 1-year OS 67%	NR
(158)AML, MRD, and lymphoma relapsed after SCT	12	Pembrolizumab	PD-1	Prospective study	Pembrolizumab 200 mg every 3 weeks for up to 2 years	AML ORR 0%	iRAEs are 42% Grade 3–4 iRAEs are 25%
(159)High-risk AML	15	Nivolumab	PD-1	Phase II, open-label, single-arm,	Nivolumab 3 mg/kg every 2 weeks for cycle 6, then nivolumab every 4 weeks for cycle 12, finally, nivolumab every 3 months until disease relapse	6-month RFS 57.1% median RFS 8.48 months	iRAEs are 40%; Grade 3–4 iRAEs are 27%
(164)R/R AML	75	Nivolumab	PD-1	Single-center retrospective cohort study	Azacitidine with nivolumab or azacitidine with nivolumab plus ipilimumab	All but 2 patients are withdrawn from the CPI trial before completion	53% experience one or more iRAEs and grade 2–3 iRAEs are the most common
(117)R/R AML	70	Nivolumab	PD-1	Phase, open-label, non-randomized	Azacitidine 75mg/m^2^ days 1–7 with nivolumab 3mg/kg on day 1 and 14, every 4–6 weeks	ORR 33% CR/CRi 22%	Grade 3–4 iRAEs are 11%
(160)AML or High-risk MDS	44	Nivolumab	PD-1	Phase, single-arm	Cytarabine 1.5 g/m² on days 1–4 and idarubicin 12 mg/m² on days 1–3. Nivolumab 3 mg/kg is started on day 24 and continues every 2 weeks for up to a year in responders	Median RFS 18.54 months mOS 18.54 months.	Grade 3–4 iRAEs are 13.6%
(161)R/R AML	19	Avelumab	PD-L1	Phase Ib/II, open-label, single-center, non-randomized	Azacitidine 75 mg/m^2^ on days 1–7 and avelumab 3 mg/kg or 10 mg/kg on days 1 and 14, every 28-day	ORR 10.5% mOS 4.8 months	Two patients experience iRAEs of grade 2 and grade 3 pneumonitis
(163)R/R AML	7	Avelumab	PD-L1	Phase Ib/II, open-label, parallel cohort	Azacitidine 75 mg/m^2^ on days 1–7, Avelumab 10 mg/kg (max dose: 2000 mg) on day 1 and day 14, gemtuzumab ozogamicin 3 mg/m^2^ (max dose: 4.5 mg) on day 8	CR 14%	Grade ≥3 iRAEs are 0%
(163)R/R AML	13	Avelumab	PD-L1	Phase Ib/II, open-label, parallel cohort	Azacitidine 75 mg/m^2^ on days 1–7, venetoclax 400 mg on days 1–28 (cycle 1)/days 1–21 (cycles 2+), avelumab 10 mg/kg (max dose: 2000 mg) on day 1 and day 14	CRi 15% mOS 4.8 months	One patient experience grade 3 iRAE
(165)AML	7	Avelumab	PD-L1	Phase I, open-label, single-arm	Decitabine 20mg/m^2^ days 1–5, every 28-day, avelumab 10mg/kg day 1, every 14-day	CR 20% mOS 3.2 months	NR
(162) Elderly AML	64	Durvalumab	PD-L1	Phase, open-label, randomized	Azacitidine 75 mg/m^2^ on days 1–7 with durvalumab 1500 mg on day 1 every 4 weeks	ORR 31.3% OS 13.0 months DOR 24.6 weeks	iRAEs. are 28.1%
(166)AML	16	Atezolizumab	PD-L1	Phase Ib, open-label, non-randomized, multicenter	Guadecitabine 60 mg/m^2^ on days 1–5 and atezolizumab 840 mg on day 8 and day 22 in 28-day cycles	87.5% patients die during the trial period due to disease progression or AEs	Grade 3–4 TRAEs of Atezolizumab are 18.8%
(167)R/R AML	11	Atezolizumab	PD-L1	Phase Ib. open-label, multicenter, non-randomized	Atezolizumab (840 mg) on day 22 of cycle 1, in subsequent 28-day cycles, atezolizumab on days 8 and 22. Magrolimab two priming doses of 1 mg/kg on days 1 and 4 of cycle 1, then 15 mg/kg on day 8, and 30 mg/kg on day 11 of cycle 1, starting on day 15, magrolimab maintenance 30 mg/kg/week.	18.2% patients withdraw from the study, and 81.8% patients die	AEs related to atezolizumab are 36.4%
(168)R/R AML	27	Tislelizumab	PD-1	Phase II, open-label, single-arm, nonrandomized	Azacitidine 75 mg/m^2^ daily, day 1–7 or decitabine 20 mg/m^2^ daily, day 1–5 plus CAG regimen (cytarabine 100 mg every 12h, day 1–5; aclarubicin 20 mg daily, day 1–5 or idarubicin 10 mg day 1, 3 and 5; and G-CSF 5 μg/kg/day, from day 0 to end) with tislelizumab 200 mg day 6 or day 8	ORR 63% CR 44% CRi 7% mOS 9.7 months EFS 9.2 months	Grade 2–3 iRAEs are 14.8%
(127)CML	31	Nivolumab	PD-1	Phase Ib	Dasatinib 100 mg (CP) or 140 mg (AP) once daily and nivolumab 0.3 mg/kg, 1 mg/kg, or 3 mg/kg every 2 weeks for ≤2 years followed by ≤1 year of dasatinib only	26% patient achieve MMR at months 12 29% patient achieve MMR at months 24	Only 2 serious AEs (both grade 2) are considered drug-related
(169)MDS and CMML	33	Atezolizumab	PD-L1	Phase I/II, multicenter	Guadecitabine 30 mg/m^2^ and escalating to 60 mg/m^2^ days 1–5, atezolizumab 840mg days 8 and 22 of a 28-day cycle.	ORR 33% mOS 15.1 months Median PFS 7.2 months	iRAEs are 36% (4 grade 3, 3 grade 2, 5 grade1)
(132) ALL and MRD	12	Pembrolizumab	PD-1	Phase	Pembrolizumab 200 mg every 3 weeks	mOS 12.7 months 8% experience a complete MRD response, which last 3 weeks	iRAEs are 8% (grade 3 Stevens-Johnson syndrome)
(140) R/R CLL	17	Pembrolizumab	PD-1	Phase Ib	Pembrolizumab 200 mg every 3 weeks plus dinaciclib 7 mg/m^2^ on day 1 and 10 mg/m^2^ on day 8 of cycle 1 and 14 mg/m^2^ on days 1 and 8 of cycles 2 and later	ORRs 29.4% median PFS 5.2 month median DOR 10.3 months mOS 21.7 months	TRAEs, any grades are 76.5%, grade 3–4 are 52.9%
(141)CLL and SLL	36	Nivolumab	PD-1	Phase I/IIa, open-label; two-part	Ibrutinib (420 mg or 560 mg) in combination with nivolumab (3 mg/kg every 2 weeks)	ORRs 61% median DOR 19.2 months The median duration of stable disease or better is 19.7 months	The most common grade 3–4 iRAEs are rash (8%) and increased ALT (2%)
(142)CLL and SLL	10	Nivolumab	PD-1	Phase II	Nivolumab 3 mg/kg every 2 weeks each 4-week cycle, starting cycle 1 day 1 for a total of 24 cycles, ibrutinib 420 mg once daily starting cycle 2 day 1	CR/CRi 30%	One patient experiences a grade 2 immunological toxicity
(170)AML relapsed after SCT	1	Tislelizumab	PD-1	Case report	Tislelizumab 100 mg on day 1 and azacitidine 100 mg on days 1–7	Achieve CR	Patient experience moderate GVHD and iRAEs
(171)AML relapsed after SCT	1	Pembrolizumab	PD-1	Case report	Pembrolizumab 100 mg	CR lasting 10 months or more	NR
(171)AML relapsed after SCT	1	Nivolumab	PD-1	Case report	Nivolumab 0.3–1 mg/kg, 5 times a week	Achieve molecular disease stabilization	NR
(171)AML relapsed after SCT	1	Nivolumab	PD-1	Case report	Nivolumab 100 mg	No objective response	NR
(172)ALL relapsed after SCT	1	Nivolumab	PD-1	Case report	Nivolumab 40 mg every 2 weeks	PET-CT show near complete resolution of pre-existing lesions, with residual low-grade metabolic uptake in the renal lesion	Owing to hepatic derangement, nivolumab is suspended
(172)ALL relapsed after SCT	1	Nivolumab	PD-1	Case report	Nivolumab 40 mg every 2 weeks for two cycles, then 80 mg every 2 weeks	Blast counts remain static for 9 weeks, but increase after the fifth dose of nivolumab	LDH and serum phosphate increase, and generalized bone pain

ICIs, immune checkpoint inhibitors; iRAE, immune-related adverse events; R/R, relapsed/refractory; AML, acute myeloid leukemia; PD-1, programmed cell death protein 1; ORR, overall response rate; CR, complete remission; CRc, composite complete remission; OS, overall survival; mOS, median overall survival; RFS, recurrence-free survival; NR, not report; MRD, measurable residual disease; SCT, stem cell transplantation; CPI, checkpoint inhibitor; CRi CR with incomplete recovery; MDS, myelodysplastic syndrome; PD-L1, programmed death-ligand 1; DOR, duration of response; AE, adverse events; TRAE, treatment-related adverse event; G-CSF, granulocyte-colony-stimulating factor; EFS, event-free survival; CML, chronic myeloid leukemia; CP, chronic Phase; AP, accelerated Phase; MMR, major molecular response; CMML, chronic myelomonocytic leukemia; PFS, progression-free survival; ALL, acute lymphoblastic leukemia; MRD, measurable residual disease; CLL, chronic lymphocytic leukemia; SLL, small lymphocytic lymphoma; ALT, alanine aminotransferase; LDH, lactate dehydrogenase.

### 6.8 Future clinical scenarios of PD-L1/PD-1 inhibitors in AML

In sum, setting of either consolidation or maintenance where, in the presence of PD-L1/PD-1 inhibitors at least partially restored immune system, they could promote measurable residual disease negativity. A very interesting therapeutic application, albeit of limited use, of checkpoint inhibitors in AML, could be in the post allo-HSCT setting, where, in the presence of AML relapse/progression, these agents might be useful in augmenting the immune reactivity of the graft, boosting the GVL effect, at the expense of also enhancing iRAEs, in combination with other chemotherapeutic drugs might improve drug sensitivity in patients with R/R AML, and in combination with other T-cell based immunotherapies such as CAR-T, BiTE, and Treg exhaustion might enhance cytotoxic responses.

## 7 Limitations of ICB in the treatment of leukemia

### 7.1 Limited efficacy of ICB treatment

Different from other preclinical studies, co-blockade of PD-1 with Tim-3 or PD-1 with TIGIT fails to restore the proliferation and degranulation of CD8+ T-cells from CLL patients (173, 174). There are many other suppressive checkpoint molecules in T cells such as A2A receptor, CD276, B7-H4, CD272, CTLA-4, LAG-3, etc. (16), and they may also play an important role in exhaustion of CD8+ T-cells. Besides, Radpour et al. (175) suggest that CD8+ T cells in AML are dysfunctional mainly due to epigenetic silencing of activating IC receptors rather than signaling by immune inhibitory IC receptors. Kalinin et al. (176) block PD-1/PD-L1 signaling in CD19 CAR-T cells by co-expression of CD19-CAR and PD-1-specific VHH domain of anti-PD-1 nanobody. Results show that although the activation of CAR-T cells with low PD-1 level increases, the survival and cytotoxicity of these cells are diminished. Functional impairment caused by disrupted PD-1 signaling is accompanied by faster maturation and upregulation of exhaustion marker TIGIT in CAR-T cells. This result proves that for prolonged CAR-T activity and successful target cell killing, the strength of activation signal provided by CAR should be balanced by negative signal from IC. It suggests simply eliminating/knocking out PD-1 is not enough if one wants to optimize CAR-T cells by disposing of negative co-stimulation. Moreover, AML is an aggressive, rapid progressive disease, which does not allow the immune system to develop a proper antileukemic response. A study shows robust antigen-specific T cell responses are generated against AML cells after localized implantation (subcutaneous), but not a systemic (intravenous) route, the latter generates a tolerant state towards the malignant cells. Which suggests the ideal scenario for promoting a leukemia-specific T cell response will likely be in the minimal residual disease setting (177). Furthermore, AML has a low mutational burden and the newly formed antigens are expressed in different other tissues of the host (16). In conclusion, there are some experiments that have not found the exact effect of PD-1 signal blocking, and the reasons for poor PD-1 efficacy are complex. This may explain why PD-1 mAbs have suboptimal clinical efficacy.

### 7.2 Adverse reactions of ICB treatment

Additionally, the application of PD-1/PD-L1 mAbs is greatly limited by adverse drug reactions during the clinical treatment of leukemia patients. Godfrey et al. (158) concluded from a prospective study that treatment with pembrolizumab after allo-SCT is feasible, but it may be associated with serious iRAEs. A case study has reported the combined use of azacitidine and tislelizumab (an PD-1 mAbs) to treat relapsed AML posttransplantation. AML patients achieve complete remission, but the patients successively develop serious iRAEs and GVHD, eventually dying from GVHD complications (178). Significant ICI-related toxicity can occur in multiple tissues and organs, such as pneumonia, glomerulonephritis, hepatitis, gastroenteritis, dermatitis, neurotoxicity, and others. Fortunately, these symptoms are usually alleviated with the prompt use of steroids (179). However, among the 75 R/R AML patients treated with nivolumab, 85% develop infections during the study period, and they are mostly severe. R/R AML patients treated with nivolumab are more likely to develop infections when treated with corticosteroids than those who are not (164). More adverse events during leukemia treatment with ICIs are shown in **Table 4**. Chemotherapy intolerance is an important cause of treatment discontinuation in leukemia patients, and reducing adverse effects during ICI therapy while aiming to improve their efficacy is equally important. Accordingly, the development of well-tolerated ICIs and the exploration of clinical protocols with few adverse effects of ICIs are the keys to solving the problem. However, given the insufficient data on the clinical application of ICIs for leukemia, further exploration is required to optimize ICI therapy.

**Table 4:** Adverse events after PD-1/PD-L1 blockade.

Study population	Antibody	Participants (n)	grade ≥3 hematological adverse events	grade ≥3 Nonhematological adverse events	solutions
(116)R/R AML	Pembrolizumab	37	Febrile neutropenia 62%; Hemolytic anemia 3%	Hypokalemia 3%; ALT increase 5%; AST increase 5%; Alkaline phosphatase increase 5%; Lymphocytic infiltration of liver 3%; Catheter-related infection 8%; Clostridium difficile colitis 3%; Hepatic infection 3%; Lung infection 26%; Typhlitis 3%; Pulmonary edema 3%; Maculopapular rash 5%	Median time to administration of systemic steroids after pembrolizumab and total duration of steroids is 15 (range, 5–23) and 14 (range, 1–35) days, respectively. iRAEs are self-limiting and fully resolve after administration of systemic steroids.
(158)AML, MRD and Lymphoma relapsed after SCT	Pembrolizumab	12	Hemolytic anemia 8%; Idiopathic thrombocytopenic purpura 8%	Fatigue 8%; Fever 17%; Pneumonitis 17%; Hyperthyroidism 8%; Secondary malignancy 8%	Steroid therapy/discontinue pembrolizumab therapy
(171)AML relapsed after SCT	Pembrolizumab	1	NR	Skin GVHD	Complete remission after 30 days with topical corticosteroids
(159)High-risk AML	Nivolumab	15	Febrile neutropenia 7%; Hemolysis 7%	ALT increase 13%; Pneumonitis 13%; Hypotension 7%; Abdominal pain 7%; Vomiting 7%; Sepsis 7%; AST increase 7%	Steroid therapy/discontinue nivolumab therapy
(160)AML or High-risk MDS	Nivolumab	44	Febrile neutropenia 32%	Nausea 2%; Diarrhoea 16%; Muscle weakness 2%; Syncope 2%; Elevated transaminases 2%; Elevated bilirubin 2%; Rash 5%; Colitis 4%; Pancreatitis 2%; Cholecystitis 2%; Small bowel obstruction 2%	All patients are treated with steroids and nivolumab interruption and are successfully re-challenged with nivolumab
(164)R/R AML	Nivolumab	75	Neutropenia 84%; Lymphopenia 79%; Combined cytopenia 71%	85% patients develop an infection during the study period, with bacterial (72%), fungal (16%), viral (11%), and parasitic (< 1%)	Infliximab/steroid therapy/antimicrobials/antibacterial
(161)R/R AML	Avelumab	19	Anemia 10.5%; Neutropenia 10.5%; Lymphopenia 5.3%	Diarrhea 5.3%; Fatigue 5.3%; Nausea 5.3%; Anorexia 5.3%; Pneumonitis 5.3%	Self-resolved/steroid therapy/anti-infective therapy/antiviral
(163)R/R AML	Avelumab	13	Febrile neutropenia 23%	Fatigue 8%; Gastrointestinal hemorrhage 8%; ALT/AST increase 8%; Increased bilirubin 8%; Infection 46%; Pericarditis 8%; Syncope 8%	NR
(165)AML	Avelumab	7	Febrile neutropenia 86%	Fatigue 14%; Weight 14%; Hypertension 57%; Edema 14%; Hypoxia 57%; Acute kidney injury 14%; Hypokalemia 29%; Oral mucositis14%; Pneumonitis 29%; Heart failure 29%	NR
(166)AML	Atezolizumab	16	Febrile neutropenia 56.3%; Anemia 18.8%; Thrombocytopenia 18.8%; Neutrophil count decrease 12.5%	Pneumonia 31.3%; Sepsis 18.8%; Hypokalemia 18.8%; Hypophosphatemia 18.8%; Failure to thrive 12.5%; Pneumonia aspiration 12.5%	NR
(167)R/R AML	Atezolizumab	11	Anemia 36.4%	Pneumonia 36.4%; Fatigue 18.2%; Hypokalemia 36.4%; Hypertension 18.2%	NR
(162)Elderly AML	Durvalumab	64	TEAEs: Thrombocytopenia 42.2%; Anemia 30%; Neutropenia 36%	TEAEs: Constipation 57.8%; imAEs: Pneumonitis 6.25%; Dermatitis 1.5%; Enteritis 1.5%; Arthritis 1.5%; Myocarditis 1.5%; Hepatitis 1.5%; Thyroiditis 1.5%; Bullous pemphigoid 1.5%; Colitis 1.5%; Progressive multifocal leukoencephalopathy 1.5%	NR
(127)CML	Nivolumab	31	Anemia 13%; Thrombocytopenia 16%; Neutropenia 16%; Febrile neutropenia 6%	Diarrhea 13%; Rash 6%; Nausea 3%; Vomiting 3%; Pyrexia 3%; Asthenia 3%	NR
(132)ALL and MRD	Pembrolizumab	12	Neutrophil count decrease 8%	Hypertension 25%; Stevens-Johnson syndrome 8%	After initiation of prednisone, all lesions resolve within days for Stevens-Johnson syndrome.
(141)CLL and SLL	Nivolumab	36	Neutropenia 53%; Anemia 25%; Thrombocytopenia 14%; Febrile neutropenia 11%	Rash 6%; Pneumonia 14%; Increased lipase 14%; Hypokalemia 8%; Increased amylase 8%; ALT increase 3%; Hypertension 6%	NR
(180)AML	Nivolumab	1	NR	PD-1 inhibitor-associated vitiligo-like depigmentation	Routine skin surveillance and no additional treatment
(181)AML relapsed after allo-SCT.	Pembrolizumab	2	NR	Pembrolizumab induce acute corneal toxicity after allo-SCT	Topical steroids, artificial tears and therapeutic soft contact lens/Topical steroids, topical serum eye drops, therapeutic soft contact lens and punctal plugs, bilateral temporary tarsorrhaphy
(182)CLL	Pembrolizumab	1	Autoimmune hemolytic anemia	NR	Prednisone/Rituximab/Ibrutinib

R/R, relapsed/refractory; AML, acute myeloid leukemia; ALT, alanine aminotransferase; AST, aspartate aminotransferase; iRAEs, immune-related adverse events; MRD, measurable residual disease; SCT, stem cell transplantation; NR, not reported; GVHD, graft versus host disease; MDS myelodysplastic syndromes; TEAEs, treatment-emergent adverse events; imAEs, immune-mediated adverse events; CML, chronic myeloid leukemia; ALL, acute lymphoblastic leukemia; CLL, chronic lymphocytic leukemia; SLL, small lymphocytic lymphoma; allo-PBSCT, allogeneic peripheral blood stem cell transplantation.

## 8 Conclusion

Blocking PD-1/PD-L1 achieves encouraging outcomes as shown by ex vivo studies and animal models, but clinical trials on PD-1/PD-L1 mAbs as single-agent in leukemia treatment show suboptimal results and varying degrees of adverse drug reactions. Fortunately, combinations of PD-1/PD-L1mAbs with other immunotherapies have shown quite promising, including the enhancement of GVL effect and reduction of GVHD in HSCT, the improvement of T-cell response in BiTE or CAR-T, and the application to multidrug chemotherapy to enhance drug sensitivity. In conclusion, ICB therapy opens new horizons for tumor immunotherapy, and future research will focus on refining combination regimens of ICIs to modulate the immune environment so that leukemia patients can maximize the benefits of ICB therapy.

## Author contributions

HC: Data curation, Investigation, Writing – original draft, Conceptualization. TW: Conceptualization, Investigation, Writing – original draft. XZ: Investigation, Writing – original draft, Data curation, Formal Analysis. SX: Conceptualization, Supervision, Writing – review & editing. HS: Supervision, Writing – review & editing, Investigation. YS: Conceptualization, Supervision, Writing – review & editing, Funding acquisition, Project administration, Resources. YL: Supervision, Writing – review & editing, Conceptualization, Funding acquisition, Project administration, Resources.
